# DIFFERENTIAL EFFECT OF SIRT1 ON MITOCHONDRIAL FUNCTION: INSIGHTS INTO MITOCHONDRIAL RESPIRATORY COMPLEXES

**DOI:** 10.1093/geroni/igae098.3652

**Published:** 2024-12-31

**Authors:** Xiaomin Zhang, Jyotsna Shrivastava, Pankaj Patyal, Ambika Verma, Shakshi Sharma, Gohar Azhar, Jeanne Y Wei

**Affiliations:** University of Arkansas for Medical Sciences, Little Rock, Arkansas, United States; University of Arkansas for Medical Sciences, little rock, Arkansas, United States; University of Arkansas for Medical Sciences, Little Rock, Arkansas, United States; University of Arkansas for Medical Sciences, Little Rock, Arkansas, United States; University of Arkansas for Medical Sciences, LITTLE ROCK, Arkansas, United States; University of Arkansas for Medical Sciences, Little Rock, Arkansas, United States; University of Arkansas for Medical Sciences, Little Rock, Arkansas, United States

## Abstract

Sirtuin-1 gene (SIRT1) regulates various cellular functions that influence metabolic homeostasis and aging. SIRT1 dysfunction has been implicated in cardiovascular diseases and mitochondrial dysfunction. However, mechanism of SIRT1 in the regulation of cellular respiration, metabolic homeostasis, and mitochondrial respiratory complexes is not well-known. We tested whether SIRT1 gene knockout (KO) in HEK293T affects mitochondrial respiratory complex function and expression profiles of associated genes compared with wildtype (WT) HEK293T cells. Oxygen consumption rate (OCR, assayed by XFe96 Analyzer and Oxygraph-O2K) was reduced (p < 0.001 and p < 0.05, respectively) in HEK293T SIRT1-KO cells compared to WT. Basal extracellular acidification rate (ECAR) was also reduced in SIRT1-KO cells relative to WT (p < 0.001), with higher levels of ATP in SIRT-1 KO (p < 0.01). SIRT1-KO significantly reduced the expression of glycolytic genes, including Phosphofructokinase P (p < 0.01), Hexokinase 2 (p < 0.05), and Phosphofructokinase Mv4 relative to WT (p < 0.05). Mitochondrial respiratory complexes are vital for ATP production through oxidative phosphorylation. Complex IV is crucial for the final step of electron transport chain. Compared to WT, significantly reduced OCR for complex IV in SIRT1-KO cells indicated mitochondrial dysfunction and impaired energy production (p < 0.05). SIRT1-KO also repressed the expression of mitochondrial respiratory complex I genes (NDUFAB1, NDUFV1, and NDUFV2), essential for efficient cellular respiration. This repression suggests compromised mitochondrial bioenergetics and metabolic homeostasis. Interestingly, SIRT1-KO also downregulated mitofusin-1, a key protein in maintaining mitochondrial morphology and size. Our data highlights that SIRT1 is crucial for regulating mitochondrial respiration, metabolism, and bioenergetics.

